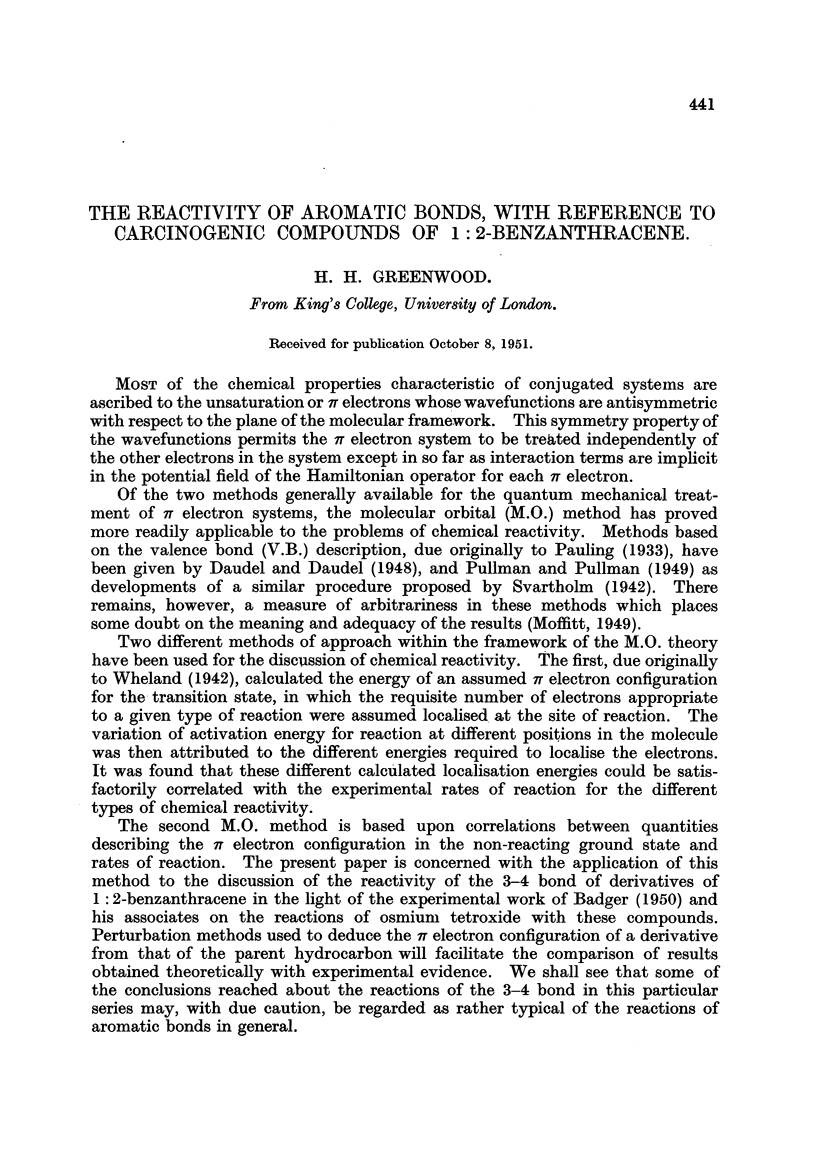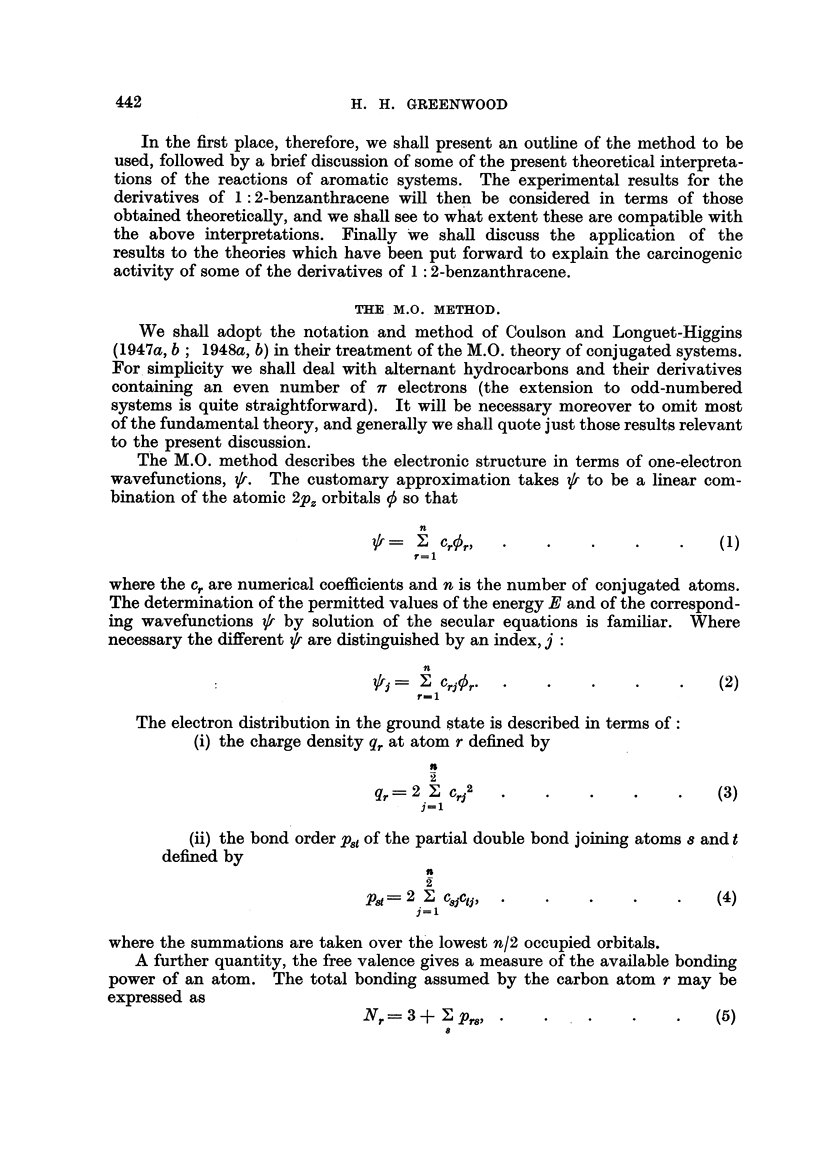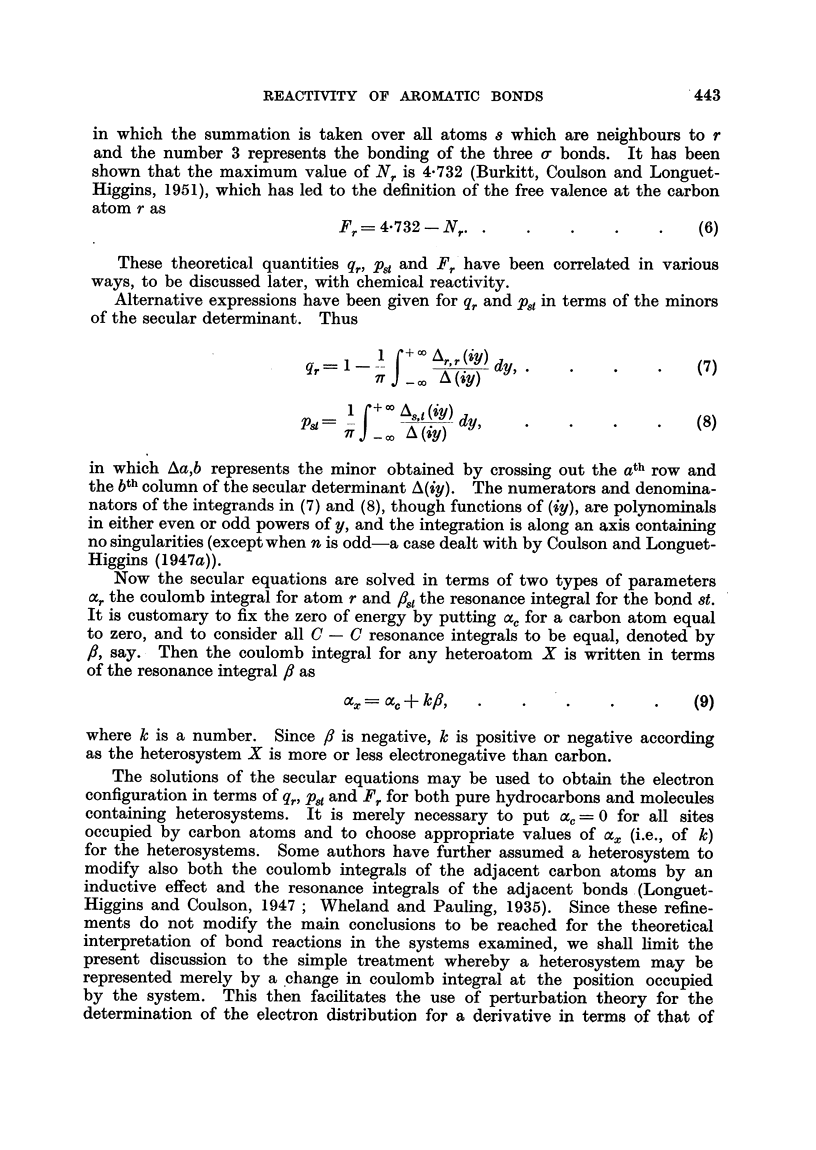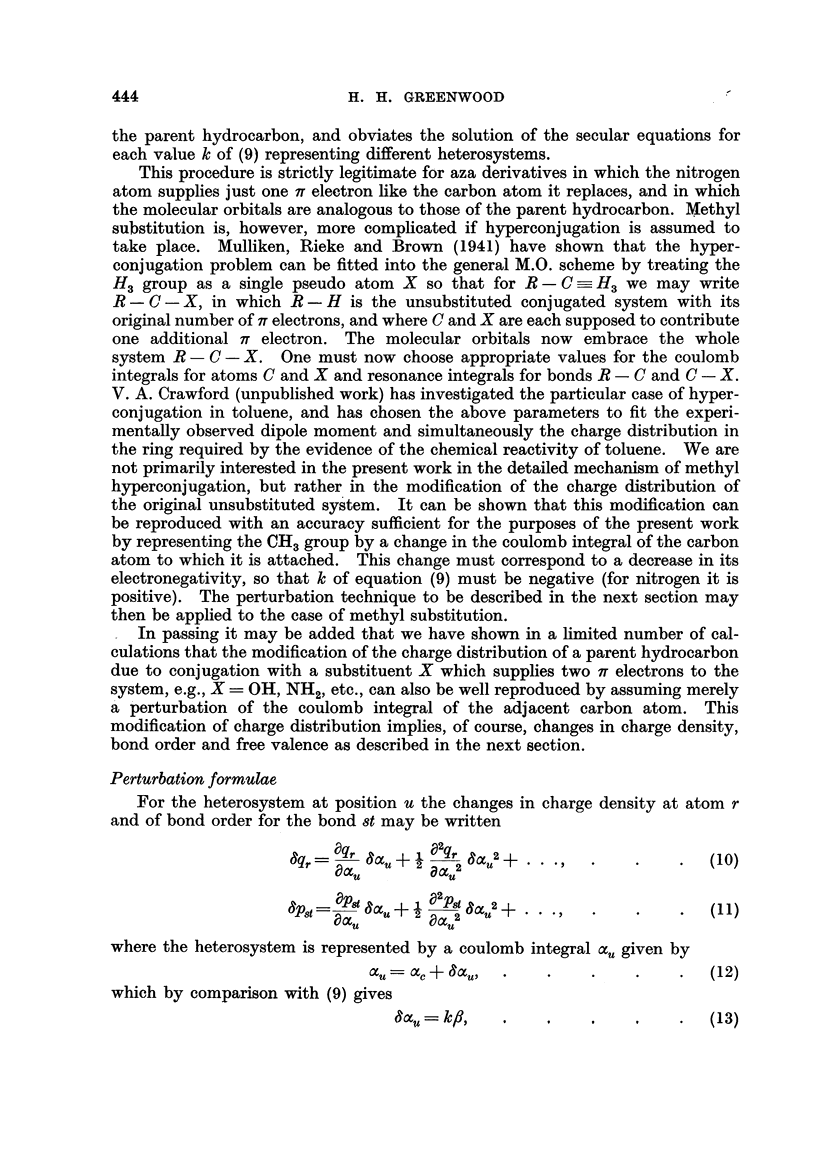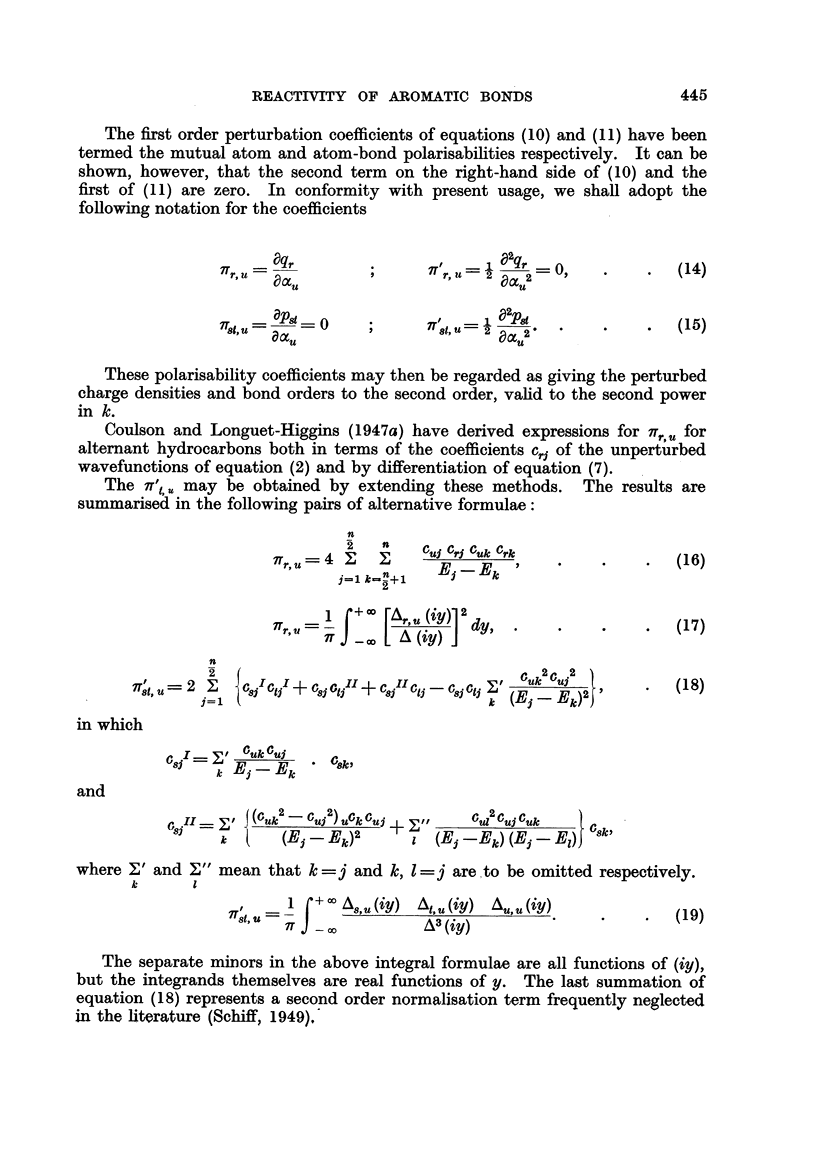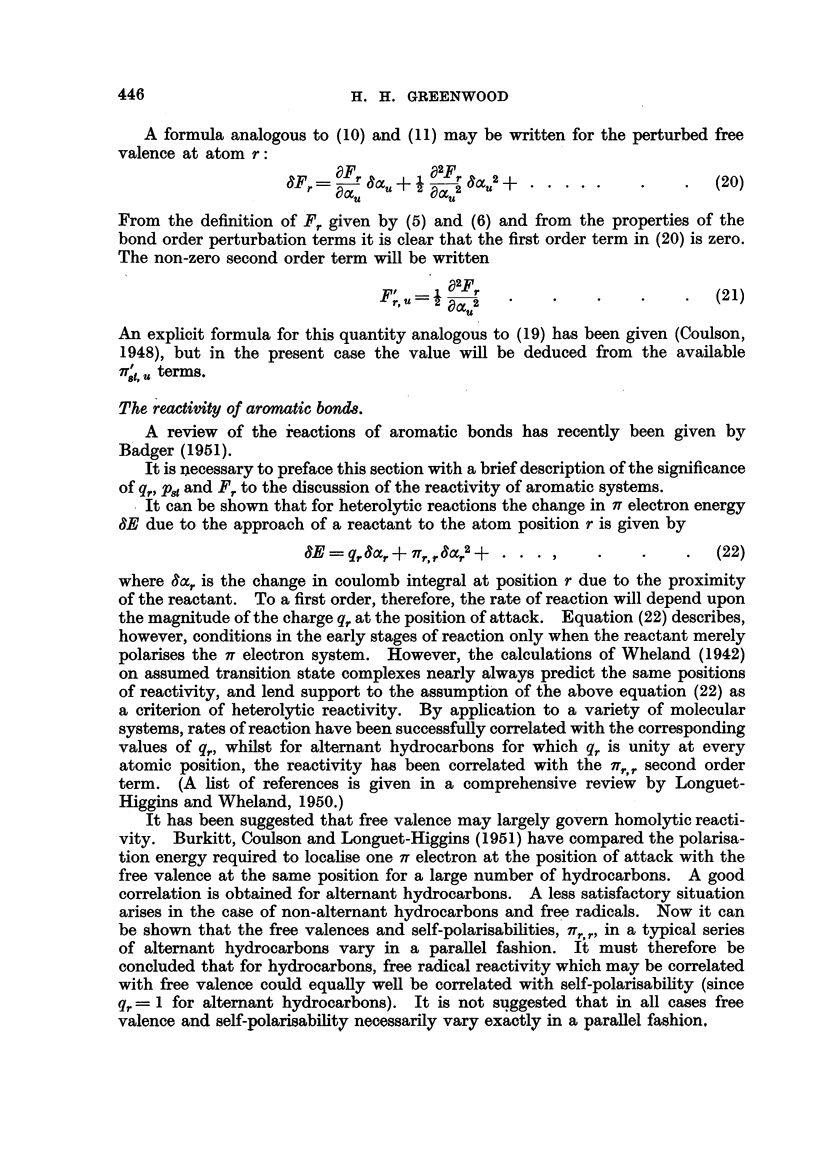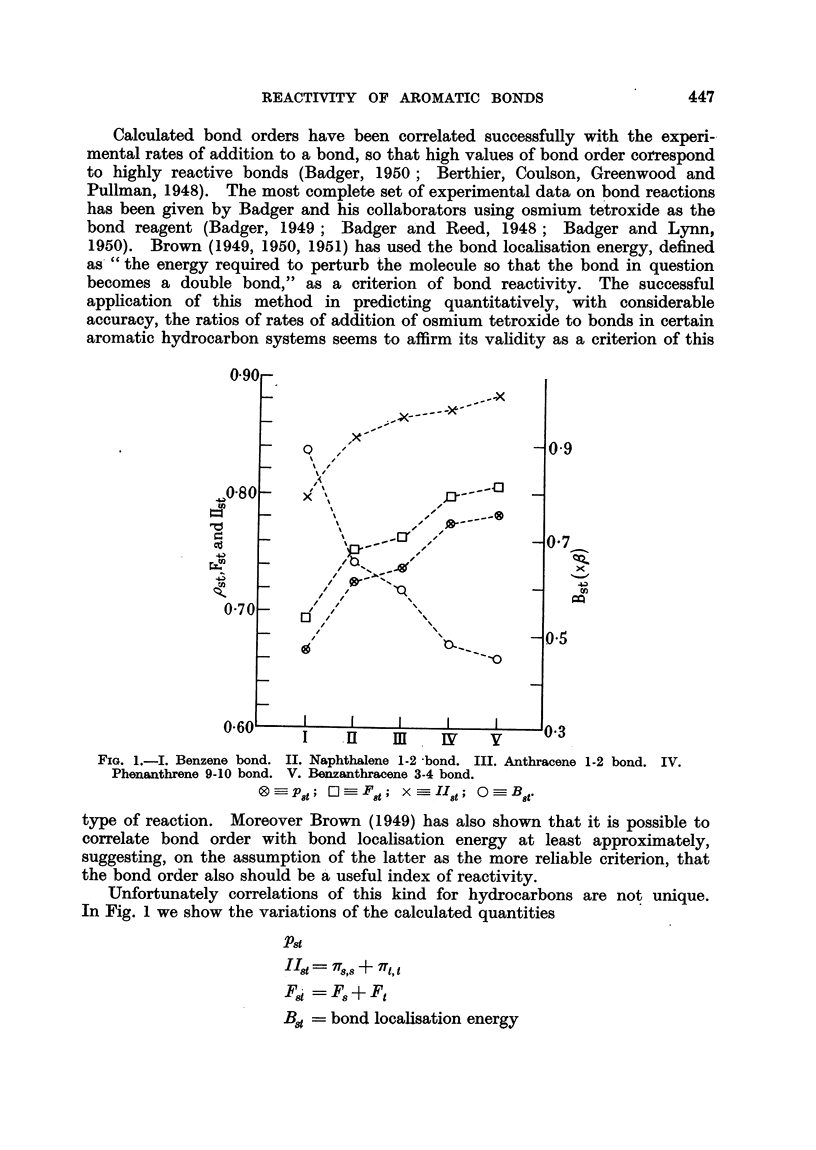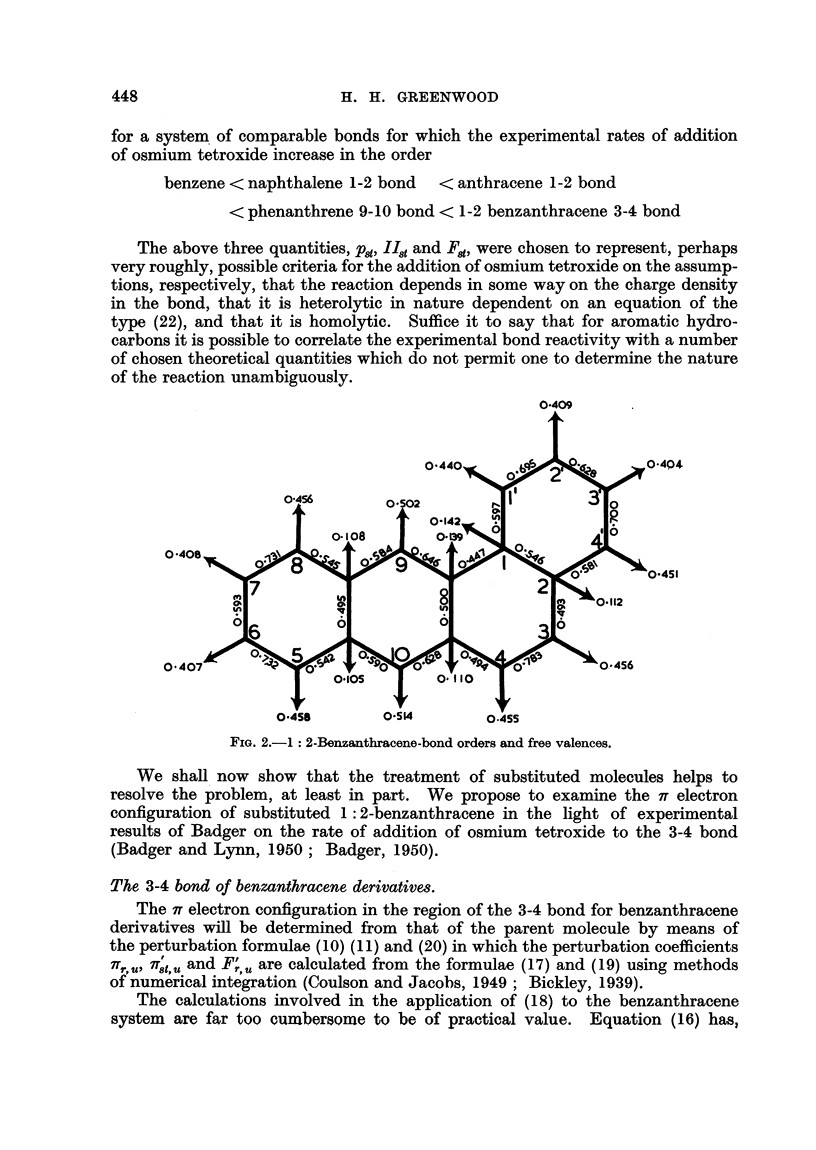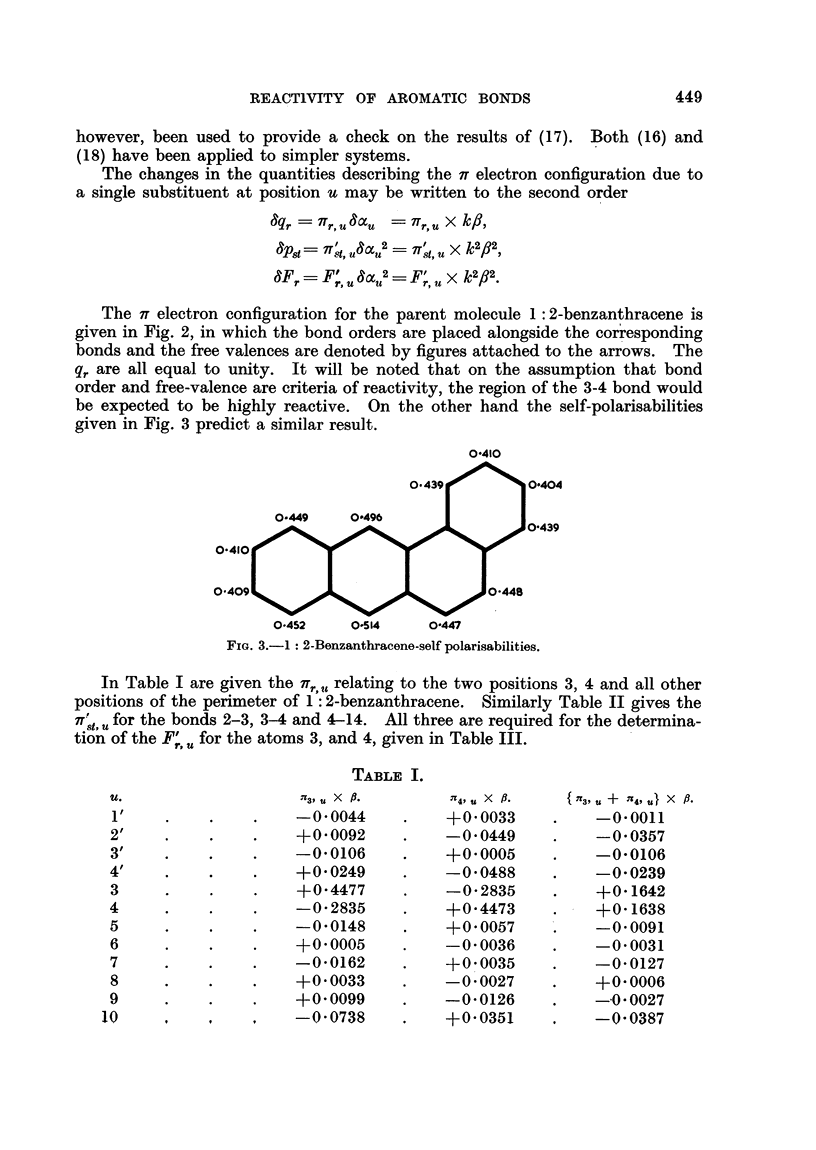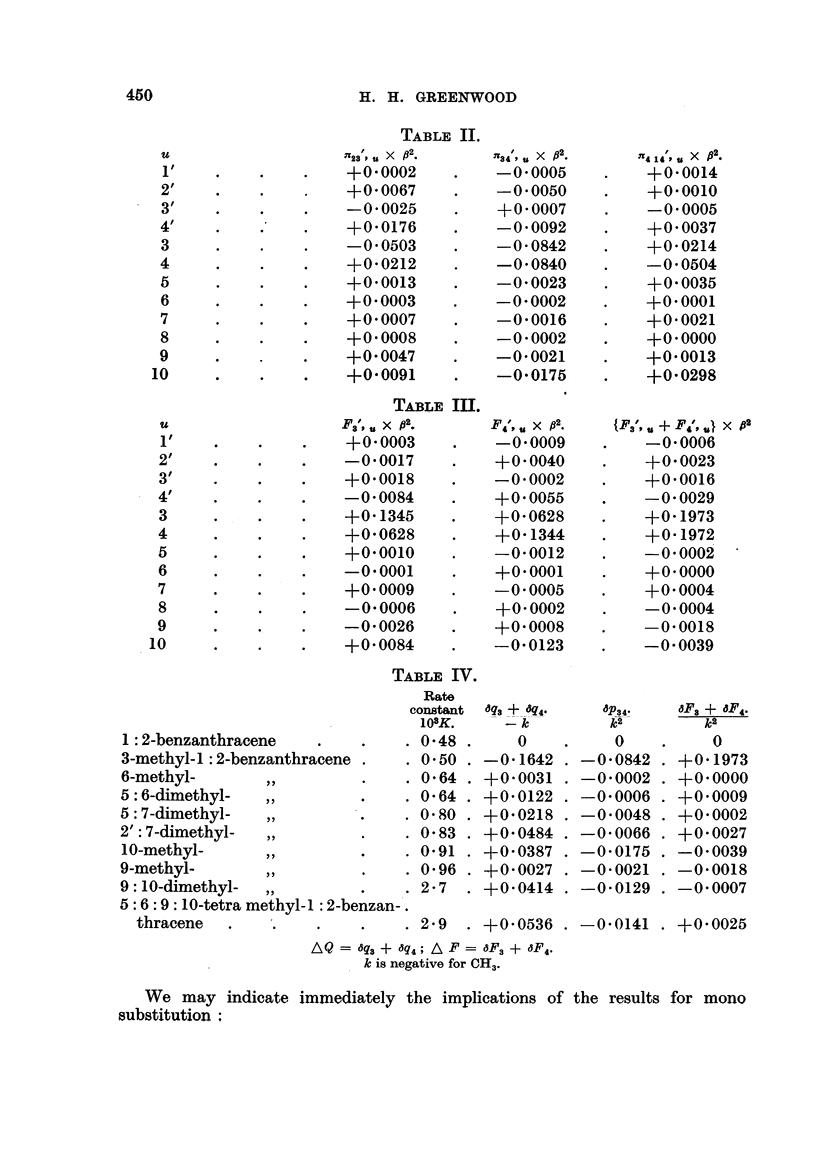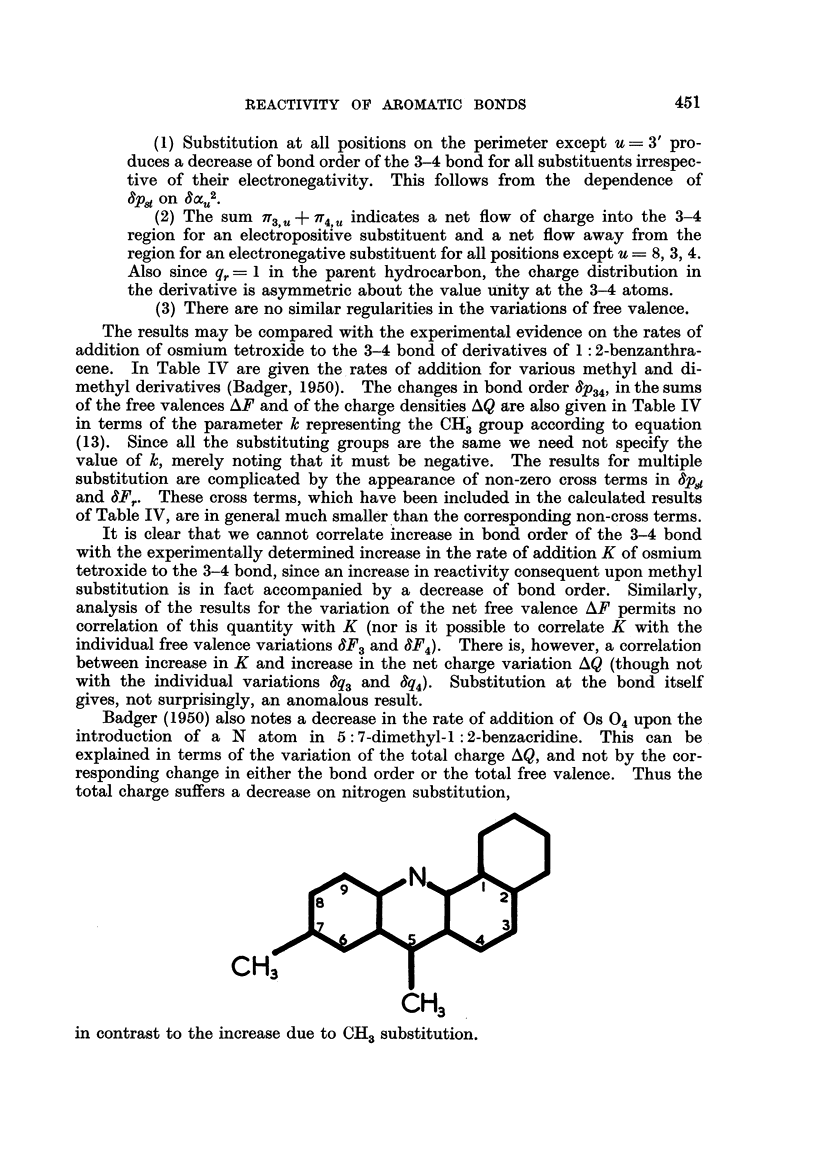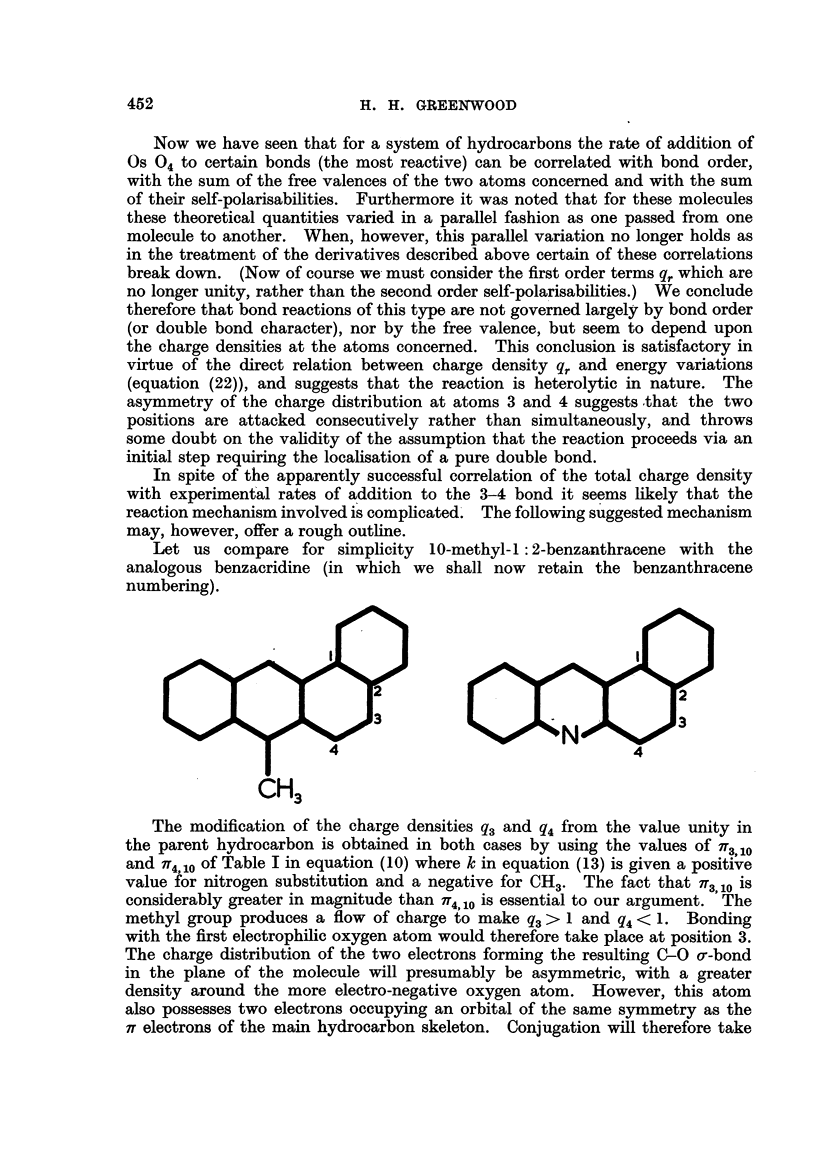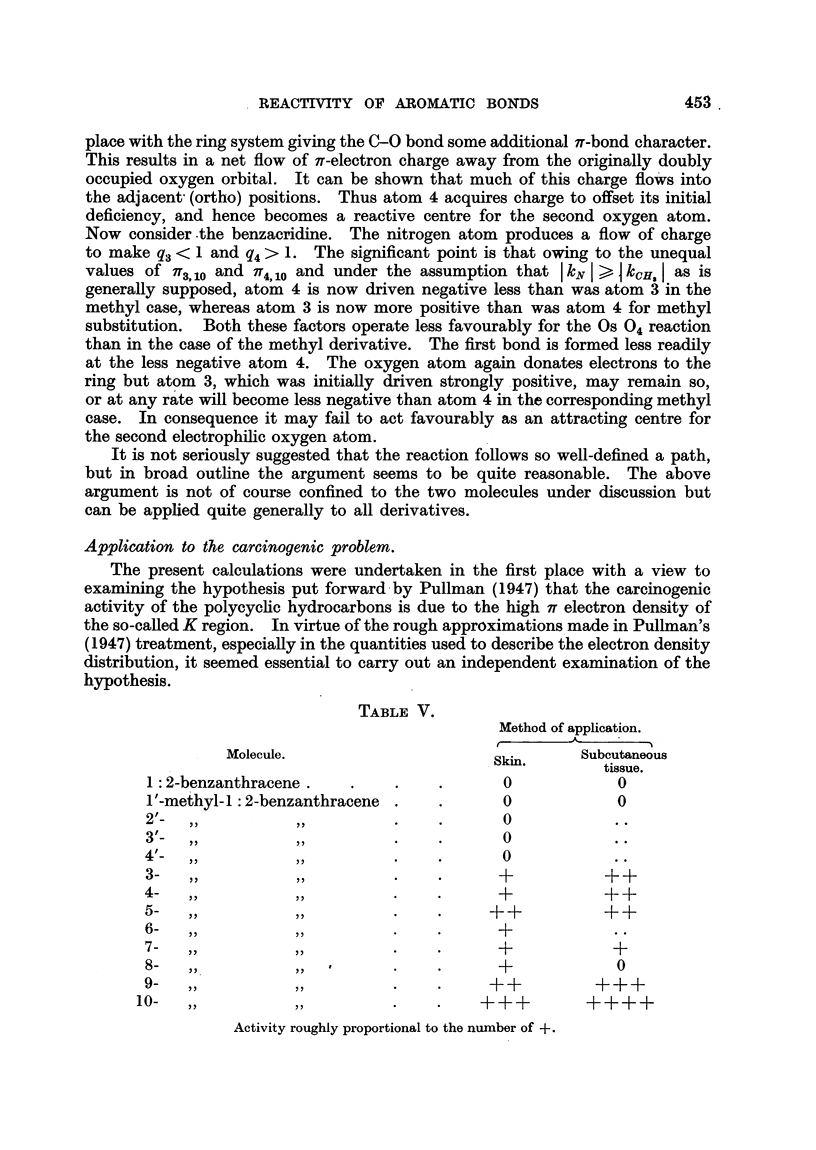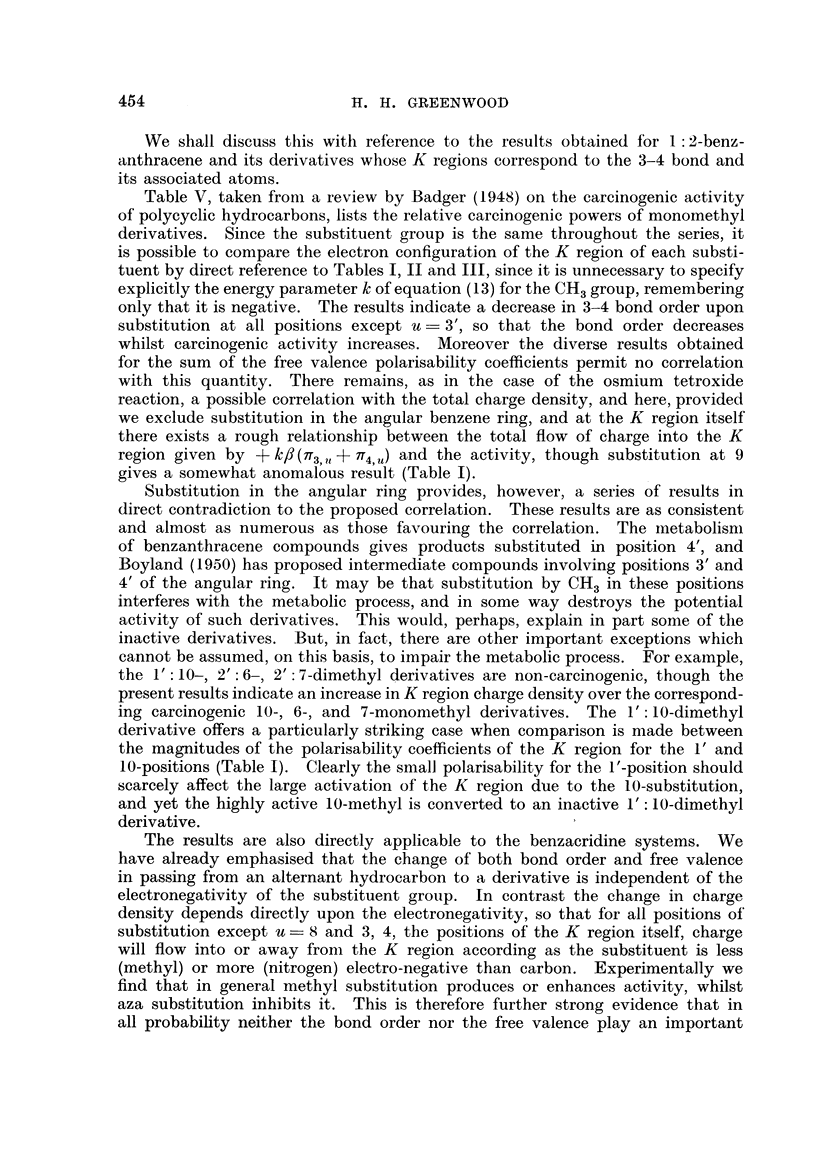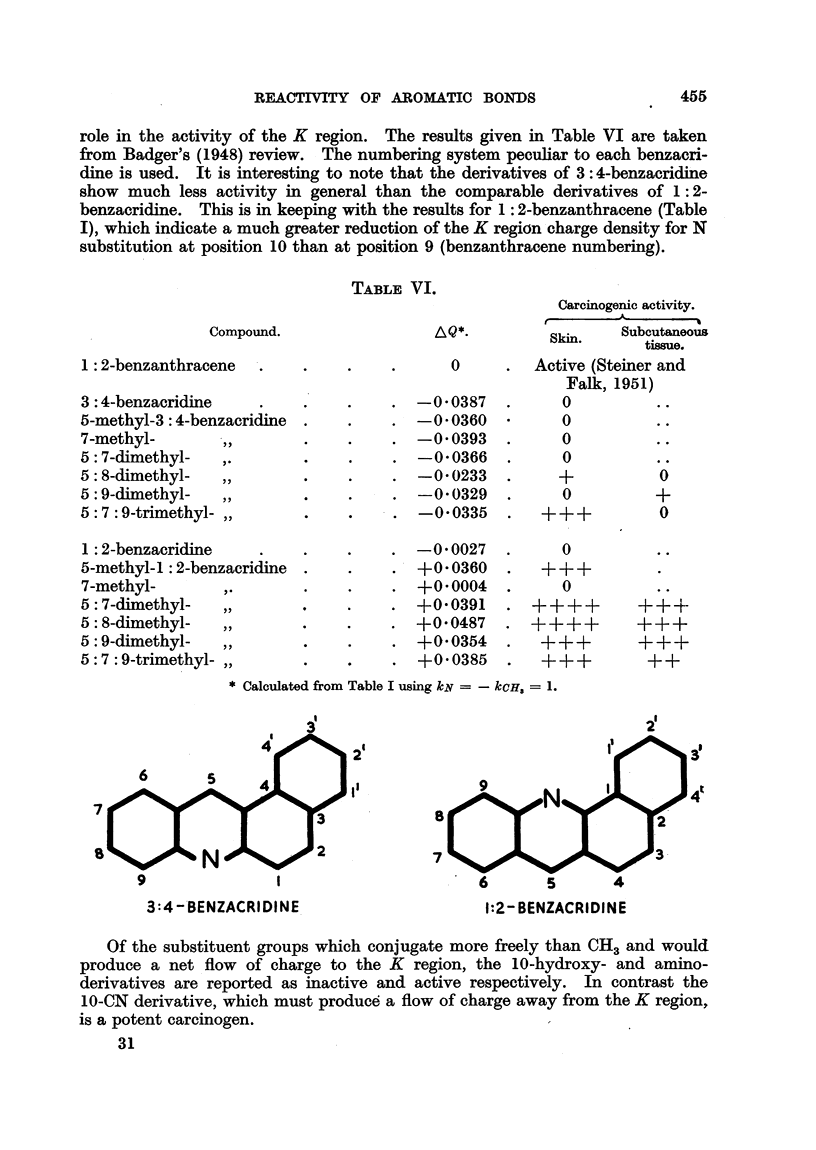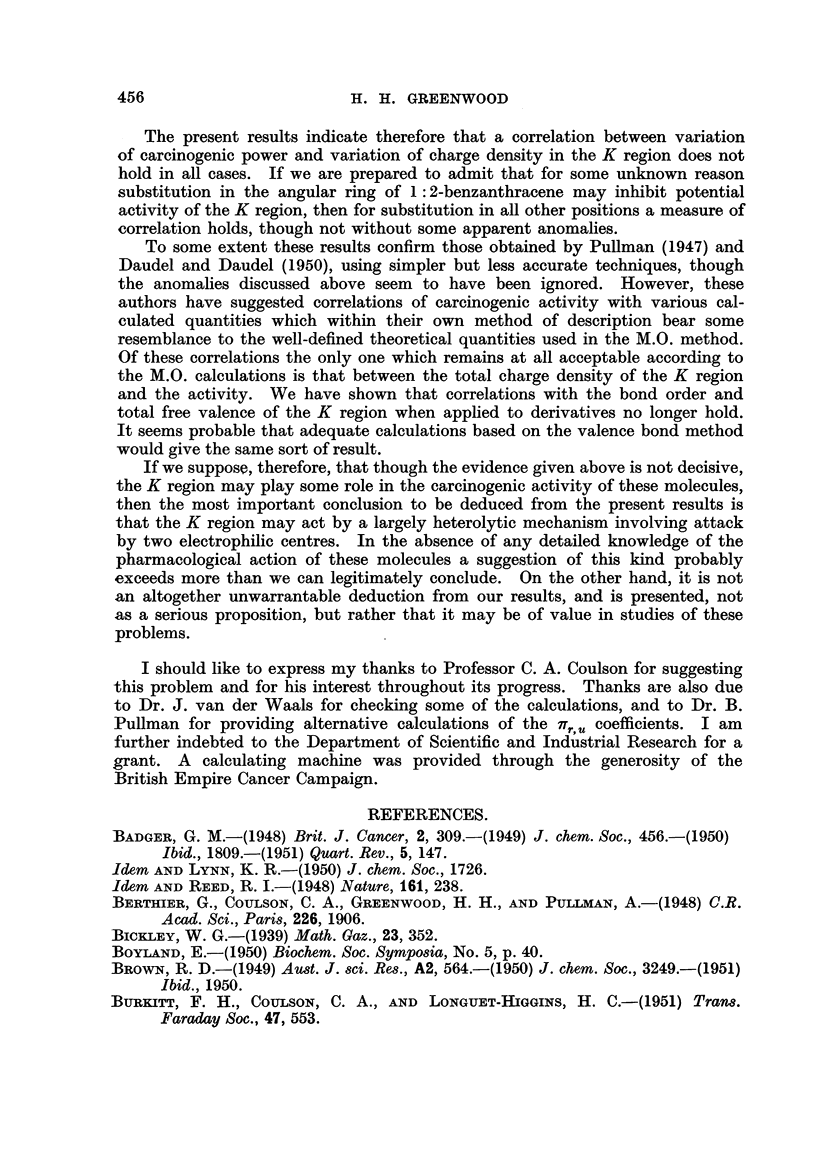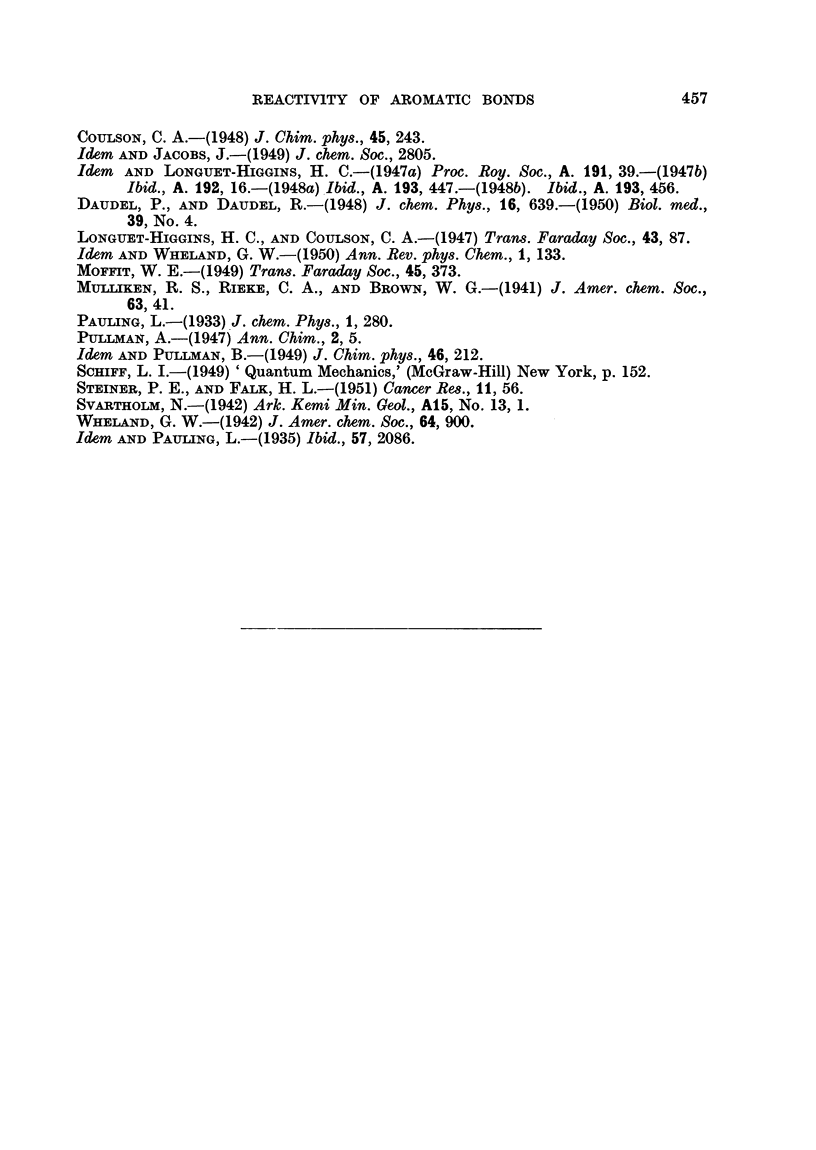# The Reactivity of Aromatic Bonds, with Reference to Carcinogenic Compounds of 1: 2-Benzanthracene

**DOI:** 10.1038/bjc.1951.50

**Published:** 1951-12

**Authors:** H. H. Greenwood


### THE REACTIVITY OF AROMATIC BONDS, WITH REFERENCE TO CARCINOGENIC COMPOUNDS OF 1 : 2-BENZANTHRACENE.

H. H. GREENWOOD.

*From King's College, University of London.*

Received for publication October 8, 1951.

MOST of the chemical properties characteristic of conjugated systems are ascribed to the unsaturation or $\pi$ electrons whose wavefunctions are antisymmetric with respect to the plane of the molecular framework. This symmetry property of the wavefunctions permits the $\pi$ electron system to be treated independently of the other electrons in the system except in so far as interaction terms are implicit in the potential field of the Hamiltonian operator for each $\pi$ electron.

Of the two methods generally available for the quantum mechanical treatment of $\pi$ electron systems, the molecular orbital (M.O.) method has proved more readily applicable to the problems of chemical reactivity. Methods based on the valence bond (V.B.) description, due originally to Pauling (1933), have been given by Daudel and Daudel (1948), and Pullman and Pullman (1949) as developments of a similar procedure proposed by Svartholm (1942). There remains, however, a measure of arbitrariness in these methods which places some doubt on the meaning and adequacy of the results (Moffitt, 1949).

Two different methods of approach within the framework of the M.O. theory have been used for the discussion of chemical reactivity. The first, due originally to Wheland (1942), calculated the energy of an assumed $\pi$ electron configuration for the transition state, in which the requisite number of electrons appropriate to a given type of reaction were assumed localised at the site of reaction. The variation of activation energy for reaction at different positions in the molecule was then attributed to the different energies required to localise the electrons. It was found that these different calculated localisation energies could be satisfactorily correlated with the experimental rates of reaction for the different types of chemical reactivity.

The second M.O. method is based upon correlations between quantities describing the $\pi$ electron configuration in the non-reacting ground state and rates of reaction. The present paper is concerned with the application of this method to the discussion of the reactivity of the 3–4 bond of derivatives of 1 : 2-benzanthracene in the light of the experimental work of Badger (1950) and his associates on the reactions of osmium tetroxide with these compounds. Perturbation methods used to deduce the $\pi$ electron configuration of a derivative from that of the parent hydrocarbon will facilitate the comparison of results obtained theoretically with experimental evidence. We shall see that some of the conclusions reached about the reactions of the 3–4 bond in this particular series may, with due caution, be regarded as rather typical of the reactions of aromatic bonds in general.

In the first place, therefore, we shall present an outline of the method to be used, followed by a brief discussion of some of the present theoretical interpretations of the reactions of aromatic systems. The experimental results for the derivatives of 1 : 2-benzanthracene will then be considered in terms of those obtained theoretically, and we shall see to what extent these are compatible with the above interpretations. Finally we shall discuss the application of the results to the theories which have been put forward to explain the carcinogenic activity of some of the derivatives of 1 : 2-benzanthracene.

### THE M.O. METHOD.

We shall adopt the notation and method of Coulson and Longuet-Higgins (1947*a*, *b* ; 1948*a*, *b*) in their treatment of the M.O. theory of conjugated systems. For simplicity we shall deal with alternant hydrocarbons and their derivatives containing an even number of $\pi$ electrons (the extension to odd-numbered systems is quite straightforward). It will be necessary moreover to omit most of the fundamental theory, and generally we shall quote just those results relevant to the present discussion.

The M.O. method describes the electronic structure in terms of one-electron wavefunctions, $\psi$. The customary approximation takes $\psi$ to be a linear combination of the atomic $2p_z$ orbitals $\phi$ so that

$$\psi = \sum_{r=1}^{n} c_r \phi_r, \qquad (1)$$

where the $c_r$ are numerical coefficients and $n$ is the number of conjugated atoms. The determination of the permitted values of the energy $E$ and of the corresponding wavefunctions $\psi$ by solution of the secular equations is familiar. Where necessary the different $\psi$ are distinguished by an index, $j$ :

$$\psi_j = \sum_{r=1}^{n} c_{rj} \phi_r. \qquad (2)$$

The electron distribution in the ground state is described in terms of :

(i) the charge density $q_r$ at atom $r$ defined by

$$q_r = 2 \sum_{j=1}^{\frac{n}{2}} c_{rj}^2 \qquad (3)$$

(ii) the bond order $p_{st}$ of the partial double bond joining atoms $s$ and $t$ defined by

$$p_{st} = 2 \sum_{j=1}^{\frac{n}{2}} c_{sj} c_{tj}, \qquad (4)$$

where the summations are taken over the lowest $n/2$ occupied orbitals.

A further quantity, the free valence gives a measure of the available bonding power of an atom. The total bonding assumed by the carbon atom $r$ may be expressed as

$$N_r = 3 + \sum_{s} p_{rs}, \qquad (5)$$

in which the summation is taken over all atoms $s$ which are neighbours to $r$ and the number 3 represents the bonding of the three $\sigma$ bonds. It has been shown that the maximum value of $N_r$ is 4·732 (Burkitt, Coulson and Longuet-Higgins, 1951), which has led to the definition of the free valence at the carbon atom $r$ as

$$F_r = 4{\cdot}732 - N_r. \qquad (6)$$

These theoretical quantities $q_r$, $p_{st}$ and $F_r$ have been correlated in various ways, to be discussed later, with chemical reactivity.

Alternative expressions have been given for $q_r$ and $p_{st}$ in terms of the minors of the secular determinant. Thus

$$q_r = 1 - \frac{1}{\pi}\int_{-\infty}^{+\infty} \frac{\Delta_{r,r}(iy)}{\Delta(iy)}\, dy, \qquad (7)$$

$$p_{st} = \frac{1}{\pi}\int_{-\infty}^{+\infty} \frac{\Delta_{s,t}(iy)}{\Delta(iy)}\, dy, \qquad (8)$$

in which $\Delta a,b$ represents the minor obtained by crossing out the $a^{\text{th}}$ row and the $b^{\text{th}}$ column of the secular determinant $\Delta(iy)$. The numerators and denominanators of the integrands in (7) and (8), though functions of $(iy)$, are polynominals in either even or odd powers of $y$, and the integration is along an axis containing no singularities (except when $n$ is odd—a case dealt with by Coulson and Longuet-Higgins (1947$a$)).

Now the secular equations are solved in terms of two types of parameters $\alpha_r$ the coulomb integral for atom $r$ and $\beta_{st}$ the resonance integral for the bond $st$. It is customary to fix the zero of energy by putting $\alpha_c$ for a carbon atom equal to zero, and to consider all $C - C$ resonance integrals to be equal, denoted by $\beta$, say. Then the coulomb integral for any heteroatom $X$ is written in terms of the resonance integral $\beta$ as

$$\alpha_x = \alpha_c + k\beta, \qquad (9)$$

where $k$ is a number. Since $\beta$ is negative, $k$ is positive or negative according as the heterosystem $X$ is more or less electronegative than carbon.

The solutions of the secular equations may be used to obtain the electron configuration in terms of $q_r$, $p_{st}$ and $F_r$ for both pure hydrocarbons and molecules containing heterosystems. It is merely necessary to put $\alpha_c = 0$ for all sites occupied by carbon atoms and to choose appropriate values of $\alpha_x$ (i.e., of $k$) for the heterosystems. Some authors have further assumed a heterosystem to modify also both the coulomb integrals of the adjacent carbon atoms by an inductive effect and the resonance integrals of the adjacent bonds (Longuet-Higgins and Coulson, 1947 ; Wheland and Pauling, 1935). Since these refinements do not modify the main conclusions to be reached for the theoretical interpretation of bond reactions in the systems examined, we shall limit the present discussion to the simple treatment whereby a heterosystem may be represented merely by a change in coulomb integral at the position occupied by the system. This then facilitates the use of perturbation theory for the determination of the electron distribution for a derivative in terms of that of the parent hydrocarbon, and obviates the solution of the secular equations for each value $k$ of (9) representing different heterosystems.

This procedure is strictly legitimate for aza derivatives in which the nitrogen atom supplies just one $\pi$ electron like the carbon atom it replaces, and in which the molecular orbitals are analogous to those of the parent hydrocarbon. Methyl substitution is, however, more complicated if hyperconjugation is assumed to take place. Mulliken, Rieke and Brown (1941) have shown that the hyperconjugation problem can be fitted into the general M.O. scheme by treating the $H_3$ group as a single pseudo atom $X$ so that for $R-C\equiv H_3$ we may write $R-C-X$, in which $R-H$ is the unsubstituted conjugated system with its original number of $\pi$ electrons, and where $C$ and $X$ are each supposed to contribute one additional $\pi$ electron. The molecular orbitals now embrace the whole system $R-C-X$. One must now choose appropriate values for the coulomb integrals for atoms $C$ and $X$ and resonance integrals for bonds $R-C$ and $C-X$. V. A. Crawford (unpublished work) has investigated the particular case of hyperconjugation in toluene, and has chosen the above parameters to fit the experimentally observed dipole moment and simultaneously the charge distribution in the ring required by the evidence of the chemical reactivity of toluene. We are not primarily interested in the present work in the detailed mechanism of methyl hyperconjugation, but rather in the modification of the charge distribution of the original unsubstituted system. It can be shown that this modification can be reproduced with an accuracy sufficient for the purposes of the present work by representing the $CH_3$ group by a change in the coulomb integral of the carbon atom to which it is attached. This change must correspond to a decrease in its electronegativity, so that $k$ of equation (9) must be negative (for nitrogen it is positive). The perturbation technique to be described in the next section may then be applied to the case of methyl substitution.

In passing it may be added that we have shown in a limited number of calculations that the modification of the charge distribution of a parent hydrocarbon due to conjugation with a substituent $X$ which supplies two $\pi$ electrons to the system, e.g., $X = OH$, $NH_2$, etc., can also be well reproduced by assuming merely a perturbation of the coulomb integral of the adjacent carbon atom. This modification of charge distribution implies, of course, changes in charge density, bond order and free valence as described in the next section.

*Perturbation formulae*

For the heterosystem at position $u$ the changes in charge density at atom $r$ and of bond order for the bond $st$ may be written

$$\delta q_r = \frac{\partial q_r}{\partial \alpha_u}\,\delta\alpha_u + \tfrac{1}{2}\,\frac{\partial^2 q_r}{\partial \alpha_u^2}\,\delta\alpha_u^2 + \ldots, \qquad (10)$$

$$\delta p_{st} = \frac{\partial p_{st}}{\partial \alpha_u}\,\delta\alpha_u + \tfrac{1}{2}\,\frac{\partial^2 p_{st}}{\partial \alpha_u^2}\,\delta\alpha_u^2 + \ldots, \qquad (11)$$

where the heterosystem is represented by a coulomb integral $\alpha_u$ given by

$$\alpha_u = \alpha_c + \delta\alpha_u, \qquad (12)$$

which by comparison with (9) gives

$$\delta\alpha_u = k\beta, \qquad (13)$$

The first order perturbation coefficients of equations (10) and (11) have been termed the mutual atom and atom-bond polarisabilities respectively. It can be shown, however, that the second term on the right-hand side of (10) and the first of (11) are zero. In conformity with present usage, we shall adopt the following notation for the coefficients

$$\pi_{r,u} = \frac{\partial q_r}{\partial \alpha_u} \quad ; \quad \pi'_{r,u} = \frac{1}{2}\frac{\partial^2 q_r}{\partial \alpha_u^2} = 0, \qquad (14)$$

$$\pi_{st,u} = \frac{\partial p_{st}}{\partial \alpha_u} = 0 \quad ; \quad \pi'_{st,u} = \frac{1}{2}\frac{\partial^2 p_{st}}{\partial \alpha_u^2}. \qquad (15)$$

These polarisability coefficients may then be regarded as giving the perturbed charge densities and bond orders to the second order, valid to the second power in $k$.

Coulson and Longuet-Higgins (1947*a*) have derived expressions for $\pi_{r,u}$ for alternant hydrocarbons both in terms of the coefficients $c_{rj}$ of the unperturbed wavefunctions of equation (2) and by differentiation of equation (7).

The $\pi'_{t,u}$ may be obtained by extending these methods. The results are summarised in the following pairs of alternative formulae:

$$\pi_{r,u} = 4 \sum_{j=1}^{\frac{n}{2}} \sum_{k=\frac{n}{2}+1}^{n} \frac{c_{uj}\, c_{rj}\, c_{uk}\, c_{rk}}{E_j - E_k}, \qquad (16)$$

$$\pi_{r,u} = \frac{1}{\pi}\int_{-\infty}^{+\infty} \left[\frac{\Delta_{r,u}(iy)}{\Delta(iy)}\right]^2 dy, \qquad (17)$$

$$\pi'_{st,u} = 2 \sum_{j=1}^{\frac{n}{2}} \left\{ c_{sj}^{I} c_{tj}^{I} + c_{sj} c_{tj}^{II} + c_{sj}^{II} c_{tj} - c_{sj} c_{tj} \sum_k{}' \frac{c_{uk}^2 c_{uj}^2}{(E_j - E_k)^2} \right\}, \qquad (18)$$

in which

$$c_{sj}^{I} = \sum_k{}' \frac{c_{uk} c_{uj}}{E_j - E_k} \cdot c_{sk},$$

and

$$c_{sj}^{II} = \sum_k{}' \left\{ \frac{(c_{uk}^2 - c_{uj}^2)_u c_k c_{uj}}{(E_j - E_k)^2} + \sum_l{}'' \frac{c_{ul}^2 c_{uj} c_{uk}}{(E_j - E_k)(E_j - E_l)} \right\} c_{sk},$$

where $\sum_k{}'$ and $\sum_l{}''$ mean that $k = j$ and $k$, $l = j$ are to be omitted respectively.

$$\pi'_{st,u} = \frac{1}{\pi}\int_{-\infty}^{+\infty} \frac{\Delta_{s,u}(iy)\ \ \Delta_{t,u}(iy)\ \ \Delta_{u,u}(iy)}{\Delta^3(iy)}. \qquad (19)$$

The separate minors in the above integral formulae are all functions of $(iy)$, but the integrands themselves are real functions of $y$. The last summation of equation (18) represents a second order normalisation term frequently neglected in the literature (Schiff, 1949).

A formula analogous to (10) and (11) may be written for the perturbed free valence at atom $r$:

$$\delta F_r = \frac{\partial F_r}{\partial \alpha_u} \delta\alpha_u + \tfrac{1}{2} \frac{\partial^2 F_r}{\partial \alpha_u^2} \delta\alpha_u^2 + \ldots \qquad (20)$$

From the definition of $F_r$ given by (5) and (6) and from the properties of the bond order perturbation terms it is clear that the first order term in (20) is zero. The non-zero second order term will be written

$$F'_{r,u} = \tfrac{1}{2} \frac{\partial^2 F_r}{\partial \alpha_u^2} \qquad (21)$$

An explicit formula for this quantity analogous to (19) has been given (Coulson, 1948), but in the present case the value will be deduced from the available $\pi'_{st,u}$ terms.

*The reactivity of aromatic bonds.*

A review of the reactions of aromatic bonds has recently been given by Badger (1951).

It is necessary to preface this section with a brief description of the significance of $q_r$, $p_{st}$ and $F_r$ to the discussion of the reactivity of aromatic systems.

It can be shown that for heterolytic reactions the change in $\pi$ electron energy $\delta E$ due to the approach of a reactant to the atom position $r$ is given by

$$\delta E = q_r \delta\alpha_r + \pi_{r,r} \delta\alpha_r^2 + \ldots, \qquad (22)$$

where $\delta\alpha_r$ is the change in coulomb integral at position $r$ due to the proximity of the reactant. To a first order, therefore, the rate of reaction will depend upon the magnitude of the charge $q_r$ at the position of attack. Equation (22) describes, however, conditions in the early stages of reaction only when the reactant merely polarises the $\pi$ electron system. However, the calculations of Wheland (1942) on assumed transition state complexes nearly always predict the same positions of reactivity, and lend support to the assumption of the above equation (22) as a criterion of heterolytic reactivity. By application to a variety of molecular systems, rates of reaction have been successfully correlated with the corresponding values of $q_r$, whilst for alternant hydrocarbons for which $q_r$ is unity at every atomic position, the reactivity has been correlated with the $\pi_{r,r}$ second order term. (A list of references is given in a comprehensive review by Longuet-Higgins and Wheland, 1950.)

It has been suggested that free valence may largely govern homolytic reactivity. Burkitt, Coulson and Longuet-Higgins (1951) have compared the polarisation energy required to localise one $\pi$ electron at the position of attack with the free valence at the same position for a large number of hydrocarbons. A good correlation is obtained for alternant hydrocarbons. A less satisfactory situation arises in the case of non-alternant hydrocarbons and free radicals. Now it can be shown that the free valences and self-polarisabilities, $\pi_{r,r}$, in a typical series of alternant hydrocarbons vary in a parallel fashion. It must therefore be concluded that for hydrocarbons, free radical reactivity which may be correlated with free valence could equally well be correlated with self-polarisability (since $q_r = 1$ for alternant hydrocarbons). It is not suggested that in all cases free valence and self-polarisability necessarily vary exactly in a parallel fashion.

Calculated bond orders have been correlated successfully with the experimental rates of addition to a bond, so that high values of bond order correspond to highly reactive bonds (Badger, 1950 ; Berthier, Coulson, Greenwood and Pullman, 1948). The most complete set of experimental data on bond reactions has been given by Badger and his collaborators using osmium tetroxide as the bond reagent (Badger, 1949 ; Badger and Reed, 1948 ; Badger and Lynn, 1950). Brown (1949, 1950, 1951) has used the bond localisation energy, defined as " the energy required to perturb the molecule so that the bond in question becomes a double bond," as a criterion of bond reactivity. The successful application of this method in predicting quantitatively, with considerable accuracy, the ratios of rates of addition of osmium tetroxide to bonds in certain aromatic hydrocarbon systems seems to affirm its validity as a criterion of this

FIG. 1.—I. Benzene bond. II. Naphthalene 1-2 bond. III. Anthracene 1-2 bond. IV. Phenanthrene 9-10 bond. V. Benzanthracene 3-4 bond.

⊗ ≡ $p_{st}$ ; □ ≡ $F_{st}$ ; × ≡ $\Pi_{st}$ ; ○ ≡ $B_{st}$.

type of reaction. Moreover Brown (1949) has also shown that it is possible to correlate bond order with bond localisation energy at least approximately, suggesting, on the assumption of the latter as the more reliable criterion, that the bond order also should be a useful index of reactivity.

Unfortunately correlations of this kind for hydrocarbons are not unique. In Fig. 1 we show the variations of the calculated quantities

$$p_{st}$$

$$\Pi_{st} = \pi_{s,s} + \pi_{t,t}$$

$$F_{st} = F_s + F_t$$

$$B_{st} = \text{bond localisation energy}$$

for a system of comparable bonds for which the experimental rates of addition of osmium tetroxide increase in the order benzene $<$ naphthalene 1-2 bond $<$ anthracene 1-2 bond

$<$ phenanthrene 9-10 bond $<$ 1-2 benzanthracene 3-4 bond

The above three quantities, $p_{st}$, $\Pi_{st}$ and $F_{st}$, were chosen to represent, perhaps very roughly, possible criteria for the addition of osmium tetroxide on the assumptions, respectively, that the reaction depends in some way on the charge density in the bond, that it is heterolytic in nature dependent on an equation of the type (22), and that it is homolytic. Suffice it to say that for aromatic hydrocarbons it is possible to correlate the experimental bond reactivity with a number of chosen theoretical quantities which do not permit one to determine the nature of the reaction unambiguously.

Fig. 2.—1 : 2-Benzanthracene-bond orders and free valences.

We shall now show that the treatment of substituted molecules helps to resolve the problem, at least in part. We propose to examine the $\pi$ electron configuration of substituted 1 : 2-benzanthracene in the light of experimental results of Badger on the rate of addition of osmium tetroxide to the 3-4 bond (Badger and Lynn, 1950 ; Badger, 1950).

*The* 3-4 *bond of benzanthracene derivatives.*

The $\pi$ electron configuration in the region of the 3-4 bond for benzanthracene derivatives will be determined from that of the parent molecule by means of the perturbation formulae (10) (11) and (20) in which the perturbation coefficients $\pi_{r,u}$, $\pi'_{st,u}$ and $F'_{r,u}$ are calculated from the formulae (17) and (19) using methods of numerical integration (Coulson and Jacobs, 1949 ; Bickley, 1939).

The calculations involved in the application of (18) to the benzanthracene system are far too cumbersome to be of practical value. Equation (16) has, however, been used to provide a check on the results of (17). Both (16) and (18) have been applied to simpler systems.

The changes in the quantities describing the $\pi$ electron configuration due to a single substituent at position $u$ may be written to the second order

$$\begin{aligned}\delta q_r &= \pi_{r,u}\,\delta\alpha_u = \pi_{r,u} \times k\beta, \\ \delta p_{st} &= \pi'_{st,u}\,\delta\alpha_u^2 = \pi'_{st,u} \times k^2\beta^2, \\ \delta F_r &= F'_{r,u}\,\delta\alpha_u^2 = F'_{r,u} \times k^2\beta^2.\end{aligned}$$

The $\pi$ electron configuration for the parent molecule 1 : 2-benzanthracene is given in Fig. 2, in which the bond orders are placed alongside the corresponding bonds and the free valences are denoted by figures attached to the arrows. The $q_r$ are all equal to unity. It will be noted that on the assumption that bond order and free-valence are criteria of reactivity, the region of the 3-4 bond would be expected to be highly reactive. On the other hand the self-polarisabilities given in Fig. 3 predict a similar result.

FIG. 3.—1 : 2-Benzanthracene-self polarisabilities.

In Table I are given the $\pi_{r,u}$ relating to the two positions 3, 4 and all other positions of the perimeter of 1 : 2-benzanthracene. Similarly Table II gives the $\pi'_{st,u}$ for the bonds 2–3, 3–4 and 4–14. All three are required for the determination of the $F'_{r,u}$ for the atoms 3, and 4, given in Table III.

TABLE I.

| $u$. | $\pi_{3,u} \times \beta$. | $\pi_{4,u} \times \beta$. | $\{\pi_{3,u} + \pi_{4,u}\} \times \beta$. |
|---|---|---|---|
| 1′ | −0·0044 | +0·0033 | −0·0011 |
| 2′ | +0·0092 | −0·0449 | −0·0357 |
| 3′ | −0·0106 | +0·0005 | −0·0106 |
| 4′ | +0·0249 | −0·0488 | −0·0239 |
| 3 | +0·4477 | −0·2835 | +0·1642 |
| 4 | −0·2835 | +0·4473 | +0·1638 |
| 5 | −0·0148 | +0·0057 | −0·0091 |
| 6 | +0·0005 | −0·0036 | −0·0031 |
| 7 | −0·0162 | +0·0035 | −0·0127 |
| 8 | +0·0033 | −0·0027 | +0·0006 |
| 9 | +0·0099 | −0·0126 | −0·0027 |
| 10 | −0·0738 | +0·0351 | −0·0387 |

TABLE II.

| $u$ | $\pi_{23'},_{u} \times \beta^2$. | $\pi_{34'},_{u} \times \beta^2$. | $\pi_{4\,14'},_{u} \times \beta^2$. |
|---|---|---|---|
| 1′ | +0·0002 | −0·0005 | +0·0014 |
| 2′ | +0·0067 | −0·0050 | +0·0010 |
| 3′ | −0·0025 | +0·0007 | −0·0005 |
| 4′ | +0·0176 | −0·0092 | +0·0037 |
| 3 | −0·0503 | −0·0842 | +0·0214 |
| 4 | +0·0212 | −0·0840 | −0·0504 |
| 5 | +0·0013 | −0·0023 | +0·0035 |
| 6 | +0·0003 | −0·0002 | +0·0001 |
| 7 | +0·0007 | −0·0016 | +0·0021 |
| 8 | +0·0008 | −0·0002 | +0·0000 |
| 9 | +0·0047 | −0·0021 | +0·0013 |
| 10 | +0·0091 | −0·0175 | +0·0298 |

TABLE III.

| $u$ | $F_{3'},_{u} \times \beta^2$. | $F_{4'},_{u} \times \beta^2$. | $\{F_{3'},_{u} + F_{4'},_{u}\} \times \beta^2$ |
|---|---|---|---|
| 1′ | +0·0003 | −0·0009 | −0·0006 |
| 2′ | −0·0017 | +0·0040 | +0·0023 |
| 3′ | +0·0018 | −0·0002 | +0·0016 |
| 4′ | −0·0084 | +0·0055 | −0·0029 |
| 3 | +0·1345 | +0·0628 | +0·1973 |
| 4 | +0·0628 | +0·1344 | +0·1972 |
| 5 | +0·0010 | −0·0012 | −0·0002 |
| 6 | −0·0001 | +0·0001 | +0·0000 |
| 7 | +0·0009 | −0·0005 | +0·0004 |
| 8 | −0·0006 | +0·0002 | −0·0004 |
| 9 | −0·0026 | +0·0008 | −0·0018 |
| 10 | +0·0084 | −0·0123 | −0·0039 |

TABLE IV.

| | Rate constant $10^3K$. | $\frac{\delta q_3 + \delta q_4}{-k}$. | $\frac{\delta p_{34}}{k^2}$. | $\frac{\delta F_3 + \delta F_4}{k^2}$. |
|---|---|---|---|---|
| 1 : 2-benzanthracene | 0·48 | 0 | 0 | 0 |
| 3-methyl-1 : 2-benzanthracene | 0·50 | −0·1642 | −0·0842 | +0·1973 |
| 6-methyl- ,, | 0·64 | +0·0031 | −0·0002 | +0·0000 |
| 5 : 6-dimethyl- ,, | 0·64 | +0·0122 | −0·0006 | +0·0009 |
| 5 : 7-dimethyl- ,, | 0·80 | +0·0218 | −0·0048 | +0·0002 |
| 2′ : 7-dimethyl- ,, | 0·83 | +0·0484 | −0·0066 | +0·0027 |
| 10-methyl- ,, | 0·91 | +0·0387 | −0·0175 | −0·0039 |
| 9-methyl- ,, | 0·96 | +0·0027 | −0·0021 | −0·0018 |
| 9 : 10-dimethyl- ,, | 2·7 | +0·0414 | −0·0129 | −0·0007 |
| 5 : 6 : 9 : 10-tetra methyl-1 : 2-benzanthracene | 2·9 | +0·0536 | −0·0141 | +0·0025 |

$\triangle Q = \delta q_3 + \delta q_4$ ; $\triangle F = \delta F_3 + \delta F_4$.

$k$ is negative for $CH_3$.

We may indicate immediately the implications of the results for mono substitution :

(1) Substitution at all positions on the perimeter except $u = 3'$ produces a decrease of bond order of the 3–4 bond for all substituents irrespective of their electronegativity. This follows from the dependence of $\delta p_{st}$ on $\delta \alpha_u^2$.

(2) The sum $\pi_{3,u} + \pi_{4,u}$ indicates a net flow of charge into the 3–4 region for an electropositive substituent and a net flow away from the region for an electronegative substituent for all positions except $u = 8, 3, 4$. Also since $q_r = 1$ in the parent hydrocarbon, the charge distribution in the derivative is asymmetric about the value unity at the 3–4 atoms.

(3) There are no similar regularities in the variations of free valence.

The results may be compared with the experimental evidence on the rates of addition of osmium tetroxide to the 3–4 bond of derivatives of 1 : 2-benzanthracene. In Table IV are given the rates of addition for various methyl and dimethyl derivatives (Badger, 1950). The changes in bond order $\delta p_{34}$, in the sums of the free valences $\Delta F$ and of the charge densities $\Delta Q$ are also given in Table IV in terms of the parameter $k$ representing the $CH_3$ group according to equation (13). Since all the substituting groups are the same we need not specify the value of $k$, merely noting that it must be negative. The results for multiple substitution are complicated by the appearance of non-zero cross terms in $\delta p_{st}$ and $\delta F_r$. These cross terms, which have been included in the calculated results of Table IV, are in general much smaller than the corresponding non-cross terms.

It is clear that we cannot correlate increase in bond order of the 3–4 bond with the experimentally determined increase in the rate of addition $K$ of osmium tetroxide to the 3–4 bond, since an increase in reactivity consequent upon methyl substitution is in fact accompanied by a decrease of bond order. Similarly, analysis of the results for the variation of the net free valence $\Delta F$ permits no correlation of this quantity with $K$ (nor is it possible to correlate $K$ with the individual free valence variations $\delta F_3$ and $\delta F_4$). There is, however, a correlation between increase in $K$ and increase in the net charge variation $\Delta Q$ (though not with the individual variations $\delta q_3$ and $\delta q_4$). Substitution at the bond itself gives, not surprisingly, an anomalous result.

Badger (1950) also notes a decrease in the rate of addition of Os $O_4$ upon the introduction of a N atom in 5 : 7-dimethyl-1 : 2-benzacridine. This can be explained in terms of the variation of the total charge $\Delta Q$, and not by the corresponding change in either the bond order or the total free valence. Thus the total charge suffers a decrease on nitrogen substitution, in contrast to the increase due to $CH_3$ substitution.

Now we have seen that for a system of hydrocarbons the rate of addition of Os $0_4$ to certain bonds (the most reactive) can be correlated with bond order, with the sum of the free valences of the two atoms concerned and with the sum of their self-polarisabilities. Furthermore it was noted that for these molecules these theoretical quantities varied in a parallel fashion as one passed from one molecule to another. When, however, this parallel variation no longer holds as in the treatment of the derivatives described above certain of these correlations break down. (Now of course we must consider the first order terms $q_r$ which are no longer unity, rather than the second order self-polarisabilities.) We conclude therefore that bond reactions of this type are not governed largely by bond order (or double bond character), nor by the free valence, but seem to depend upon the charge densities at the atoms concerned. This conclusion is satisfactory in virtue of the direct relation between charge density $q_r$ and energy variations (equation (22)), and suggests that the reaction is heterolytic in nature. The asymmetry of the charge distribution at atoms 3 and 4 suggests that the two positions are attacked consecutively rather than simultaneously, and throws some doubt on the validity of the assumption that the reaction proceeds via an initial step requiring the localisation of a pure double bond.

In spite of the apparently successful correlation of the total charge density with experimental rates of addition to the 3–4 bond it seems likely that the reaction mechanism involved is complicated. The following suggested mechanism may, however, offer a rough outline.

Let us compare for simplicity 10-methyl-1 : 2-benzanthracene with the analogous benzacridine (in which we shall now retain the benzanthracene numbering).

The modification of the charge densities $q_3$ and $q_4$ from the value unity in the parent hydrocarbon is obtained in both cases by using the values of $\pi_{3,10}$ and $\pi_{4,10}$ of Table I in equation (10) where $k$ in equation (13) is given a positive value for nitrogen substitution and a negative for $CH_3$. The fact that $\pi_{3,10}$ is considerably greater in magnitude than $\pi_{4,10}$ is essential to our argument. The methyl group produces a flow of charge to make $q_3 > 1$ and $q_4 < 1$. Bonding with the first electrophilic oxygen atom would therefore take place at position 3. The charge distribution of the two electrons forming the resulting C–O $\sigma$-bond in the plane of the molecule will presumably be asymmetric, with a greater density around the more electro-negative oxygen atom. However, this atom also possesses two electrons occupying an orbital of the same symmetry as the $\pi$ electrons of the main hydrocarbon skeleton. Conjugation will therefore take place with the ring system giving the C–O bond some additional $\pi$-bond character. This results in a net flow of $\pi$-electron charge away from the originally doubly occupied oxygen orbital. It can be shown that much of this charge flows into the adjacent (ortho) positions. Thus atom 4 acquires charge to offset its initial deficiency, and hence becomes a reactive centre for the second oxygen atom. Now consider the benzacridine. The nitrogen atom produces a flow of charge to make $q_3 < 1$ and $q_4 > 1$. The significant point is that owing to the unequal values of $\pi_{3,10}$ and $\pi_{4,10}$ and under the assumption that $|k_N| \geqslant |k_{CH_3}|$ as is generally supposed, atom 4 is now driven negative less than was atom 3 in the methyl case, whereas atom 3 is now more positive than was atom 4 for methyl substitution. Both these factors operate less favourably for the $OsO_4$ reaction than in the case of the methyl derivative. The first bond is formed less readily at the less negative atom 4. The oxygen atom again donates electrons to the ring but atom 3, which was initially driven strongly positive, may remain so, or at any rate will become less negative than atom 4 in the corresponding methyl case. In consequence it may fail to act favourably as an attracting centre for the second electrophilic oxygen atom.

It is not seriously suggested that the reaction follows so well-defined a path, but in broad outline the argument seems to be quite reasonable. The above argument is not of course confined to the two molecules under discussion but can be applied quite generally to all derivatives.

*Application to the carcinogenic problem.*

The present calculations were undertaken in the first place with a view to examining the hypothesis put forward by Pullman (1947) that the carcinogenic activity of the polycyclic hydrocarbons is due to the high $\pi$ electron density of the so-called $K$ region. In virtue of the rough approximations made in Pullman's (1947) treatment, especially in the quantities used to describe the electron density distribution, it seemed essential to carry out an independent examination of the hypothesis.

TABLE V.

| Molecule. | Method of application. Skin. | Subcutaneous tissue. |
|---|---|---|
| 1 : 2-benzanthracene | 0 | 0 |
| 1′-methyl-1 : 2-benzanthracene | 0 | 0 |
| 2′- ,, ,, | 0 | .. |
| 3′- ,, ,, | 0 | .. |
| 4′- ,, ,, | 0 | .. |
| 3- ,, ,, | + | ++ |
| 4- ,, ,, | + | ++ |
| 5- ,, ,, | ++ | ++ |
| 6- ,, ,, | + | .. |
| 7- ,, ,, | + | + |
| 8- ,, ,, | + | 0 |
| 9- ,, ,, | ++ | +++ |
| 10- ,, ,, | +++ | ++++ |

Activity roughly proportional to the number of +.

We shall discuss this with reference to the results obtained for 1 : 2-benzanthracene and its derivatives whose $K$ regions correspond to the 3–4 bond and its associated atoms.

Table V, taken from a review by Badger (1948) on the carcinogenic activity of polycyclic hydrocarbons, lists the relative carcinogenic powers of monomethyl derivatives. Since the substituent group is the same throughout the series, it is possible to compare the electron configuration of the $K$ region of each substituent by direct reference to Tables I, II and III, since it is unnecessary to specify explicitly the energy parameter $k$ of equation (13) for the $CH_3$ group, remembering only that it is negative. The results indicate a decrease in 3–4 bond order upon substitution at all positions except $u = 3'$, so that the bond order decreases whilst carcinogenic activity increases. Moreover the diverse results obtained for the sum of the free valence polarisability coefficients permit no correlation with this quantity. There remains, as in the case of the osmium tetroxide reaction, a possible correlation with the total charge density, and here, provided we exclude substitution in the angular benzene ring, and at the $K$ region itself there exists a rough relationship between the total flow of charge into the $K$ region given by $+k\beta(\pi_{3,u} + \pi_{4,u})$ and the activity, though substitution at 9 gives a somewhat anomalous result (Table I).

Substitution in the angular ring provides, however, a series of results in direct contradiction to the proposed correlation. These results are as consistent and almost as numerous as those favouring the correlation. The metabolism of benzanthracene compounds gives products substituted in position 4′, and Boyland (1950) has proposed intermediate compounds involving positions 3′ and 4′ of the angular ring. It may be that substitution by $CH_3$ in these positions interferes with the metabolic process, and in some way destroys the potential activity of such derivatives. This would, perhaps, explain in part some of the inactive derivatives. But, in fact, there are other important exceptions which cannot be assumed, on this basis, to impair the metabolic process. For example, the 1′ : 10–, 2′ : 6–, 2′ : 7-dimethyl derivatives are non-carcinogenic, though the present results indicate an increase in $K$ region charge density over the corresponding carcinogenic 10-, 6-, and 7-monomethyl derivatives. The 1′ : 10-dimethyl derivative offers a particularly striking case when comparison is made between the magnitudes of the polarisability coefficients of the $K$ region for the 1′ and 10-positions (Table I). Clearly the small polarisability for the 1′-position should scarcely affect the large activation of the $K$ region due to the 10-substitution, and yet the highly active 10-methyl is converted to an inactive 1′ : 10-dimethyl derivative.

The results are also directly applicable to the benzacridine systems. We have already emphasised that the change of both bond order and free valence in passing from an alternant hydrocarbon to a derivative is independent of the electronegativity of the substituent group. In contrast the change in charge density depends directly upon the electronegativity, so that for all positions of substitution except $u = 8$ and 3, 4, the positions of the $K$ region itself, charge will flow into or away from the $K$ region according as the substituent is less (methyl) or more (nitrogen) electro-negative than carbon. Experimentally we find that in general methyl substitution produces or enhances activity, whilst aza substitution inhibits it. This is therefore further strong evidence that in all probability neither the bond order nor the free valence play an important role in the activity of the *K* region. The results given in Table VI are taken from Badger's (1948) review. The numbering system peculiar to each benzacridine is used. It is interesting to note that the derivatives of 3 : 4-benzacridine show much less activity in general than the comparable derivatives of 1 : 2-benzacridine. This is in keeping with the results for 1 : 2-benzanthracene (Table I), which indicate a much greater reduction of the *K* region charge density for N substitution at position 10 than at position 9 (benzanthracene numbering).

Table VI.

| Compound. | $\Delta Q$*. | Carcinogenic activity. Skin. | Subcutaneous tissue. |
|---|---|---|---|
| 1 : 2-benzanthracene | 0 | Active (Steiner and Falk, 1951) | |
| 3 : 4-benzacridine | −0·0387 | 0 | .. |
| 5-methyl-3 : 4-benzacridine | −0·0360 | 0 | .. |
| 7-methyl- ,, | −0·0393 | 0 | .. |
| 5 : 7-dimethyl- ,, | −0·0366 | 0 | .. |
| 5 : 8-dimethyl- ,, | −0·0233 | + | 0 |
| 5 : 9-dimethyl- ,, | −0·0329 | 0 | + |
| 5 : 7 : 9-trimethyl- ,, | −0·0335 | +++ | 0 |
| 1 : 2-benzacridine | −0·0027 | 0 | .. |
| 5-methyl-1 : 2-benzacridine | +0·0360 | +++ | . |
| 7-methyl- ,, | +0·0004 | 0 | .. |
| 5 : 7-dimethyl- ,, | +0·0391 | ++++ | +++ |
| 5 : 8-dimethyl- ,, | +0·0487 | ++++ | +++ |
| 5 : 9-dimethyl- ,, | +0·0354 | +++ | +++ |
| 5 : 7 : 9-trimethyl- ,, | +0·0385 | +++ | ++ |

* Calculated from Table I using $k_N = -k_{CH_3} = 1$.

3:4-BENZACRIDINE

1:2-BENZACRIDINE

Of the substituent groups which conjugate more freely than $CH_3$ and would produce a net flow of charge to the *K* region, the 10-hydroxy- and amino-derivatives are reported as inactive and active respectively. In contrast the 10-CN derivative, which must produce a flow of charge away from the *K* region, is a potent carcinogen.

The present results indicate therefore that a correlation between variation of carcinogenic power and variation of charge density in the *K* region does not hold in all cases. If we are prepared to admit that for some unknown reason substitution in the angular ring of 1 : 2-benzanthracene may inhibit potential activity of the *K* region, then for substitution in all other positions a measure of correlation holds, though not without some apparent anomalies.

To some extent these results confirm those obtained by Pullman (1947) and Daudel and Daudel (1950), using simpler but less accurate techniques, though the anomalies discussed above seem to have been ignored. However, these authors have suggested correlations of carcinogenic activity with various calculated quantities which within their own method of description bear some resemblance to the well-defined theoretical quantities used in the M.O. method. Of these correlations the only one which remains at all acceptable according to the M.O. calculations is that between the total charge density of the *K* region and the activity. We have shown that correlations with the bond order and total free valence of the *K* region when applied to derivatives no longer hold. It seems probable that adequate calculations based on the valence bond method would give the same sort of result.

If we suppose, therefore, that though the evidence given above is not decisive, the *K* region may play some role in the carcinogenic activity of these molecules, then the most important conclusion to be deduced from the present results is that the *K* region may act by a largely heterolytic mechanism involving attack by two electrophilic centres. In the absence of any detailed knowledge of the pharmacological action of these molecules a suggestion of this kind probably exceeds more than we can legitimately conclude. On the other hand, it is not an altogether unwarrantable deduction from our results, and is presented, not as a serious proposition, but rather that it may be of value in studies of these problems.

I should like to express my thanks to Professor C. A. Coulson for suggesting this problem and for his interest throughout its progress. Thanks are also due to Dr. J. van der Waals for checking some of the calculations, and to Dr. B. Pullman for providing alternative calculations of the $\pi_{r,u}$ coefficients. I am further indebted to the Department of Scientific and Industrial Research for a grant. A calculating machine was provided through the generosity of the British Empire Cancer Campaign.

### REFERENCES.

BADGER, G. M.—(1948) *Brit. J. Cancer*, **2**, 309.—(1949) *J. chem. Soc.*, 456.—(1950) *Ibid.*, 1809.—(1951) *Quart. Rev.*, **5**, 147.

*Idem* AND LYNN, K. R.—(1950) *J. chem. Soc.*, 1726.

*Idem* AND REED, R. I.—(1948) *Nature*, **161**, 238.

BERTHIER, G., COULSON, C. A., GREENWOOD, H. H., AND PULLMAN, A.—(1948) *C.R. Acad. Sci., Paris*, **226**, 1906.

BICKLEY, W. G.—(1939) *Math. Gaz.*, **23**, 352.

BOYLAND, E.—(1950) *Biochem. Soc. Symposia*, No. 5, p. 40.

BROWN, R. D.—(1949) *Aust. J. sci. Res.*, **A2**, 564.—(1950) *J. chem. Soc.*, 3249.—(1951) *Ibid.*, 1950.

BURKITT, F. H., COULSON, C. A., AND LONGUET-HIGGINS, H. C.—(1951) *Trans. Faraday Soc.*, **47**, 553.

COULSON, C. A.—(1948) *J. Chim. phys.*, **45**, 243.
*Idem* AND JACOBS, J.—(1949) *J. chem. Soc.*, 2805.
*Idem* AND LONGUET-HIGGINS, H. C.—(1947*a*) *Proc. Roy. Soc.*, **A. 191**, 39.—(1947*b*) *Ibid.*, **A. 192**, 16.—(1948*a*) *Ibid.*, **A. 193**, 447.—(1948*b*). *Ibid.*, **A. 193**, 456.
DAUDEL, P., AND DAUDEL, R.—(1948) *J. chem. Phys.*, **16**, 639.—(1950) *Biol. med.*, **39**, No. 4.
LONGUET-HIGGINS, H. C., AND COULSON, C. A.—(1947) *Trans. Faraday Soc.*, **43**, 87.
*Idem* AND WHELAND, G. W.—(1950) *Ann. Rev. phys. Chem.*, **1**, 133.
MOFFIT, W. E.—(1949) *Trans. Faraday Soc.*, **45**, 373.
MULLIKEN, R. S., RIEKE, C. A., AND BROWN, W. G.—(1941) *J. Amer. chem. Soc.*, **63**, 41.
PAULING, L.—(1933) *J. chem. Phys.*, **1**, 280.
PULLMAN, A.—(1947) *Ann. Chim.*, **2**, 5.
*Idem* AND PULLMAN, B.—(1949) *J. Chim. phys.*, **46**, 212.
SCHIFF, L. I.—(1949) ‘ Quantum Mechanics,’ (McGraw-Hill) New York, p. 152.
STEINER, P. E., AND FALK, H. L.—(1951) *Cancer Res.*, **11**, 56.
SVARTHOLM, N.—(1942) *Ark. Kemi Min. Geol.*, **A15**, No. 13, 1.
WHELAND, G. W.—(1942) *J. Amer. chem. Soc.*, **64**, 900.
*Idem* AND PAULING, L.—(1935) *Ibid.*, **57**, 2086.